# Supplementary figures and images for: Live imaging of Yersinia translocon formation and immune recognition in host cells
Source: PLoS Pathog. 2022 May 23;18(5):e1010251. doi: 10.1371/journal.ppat.1010251 (PMC9173619; doi:10.1371/journal.ppat.1010251)

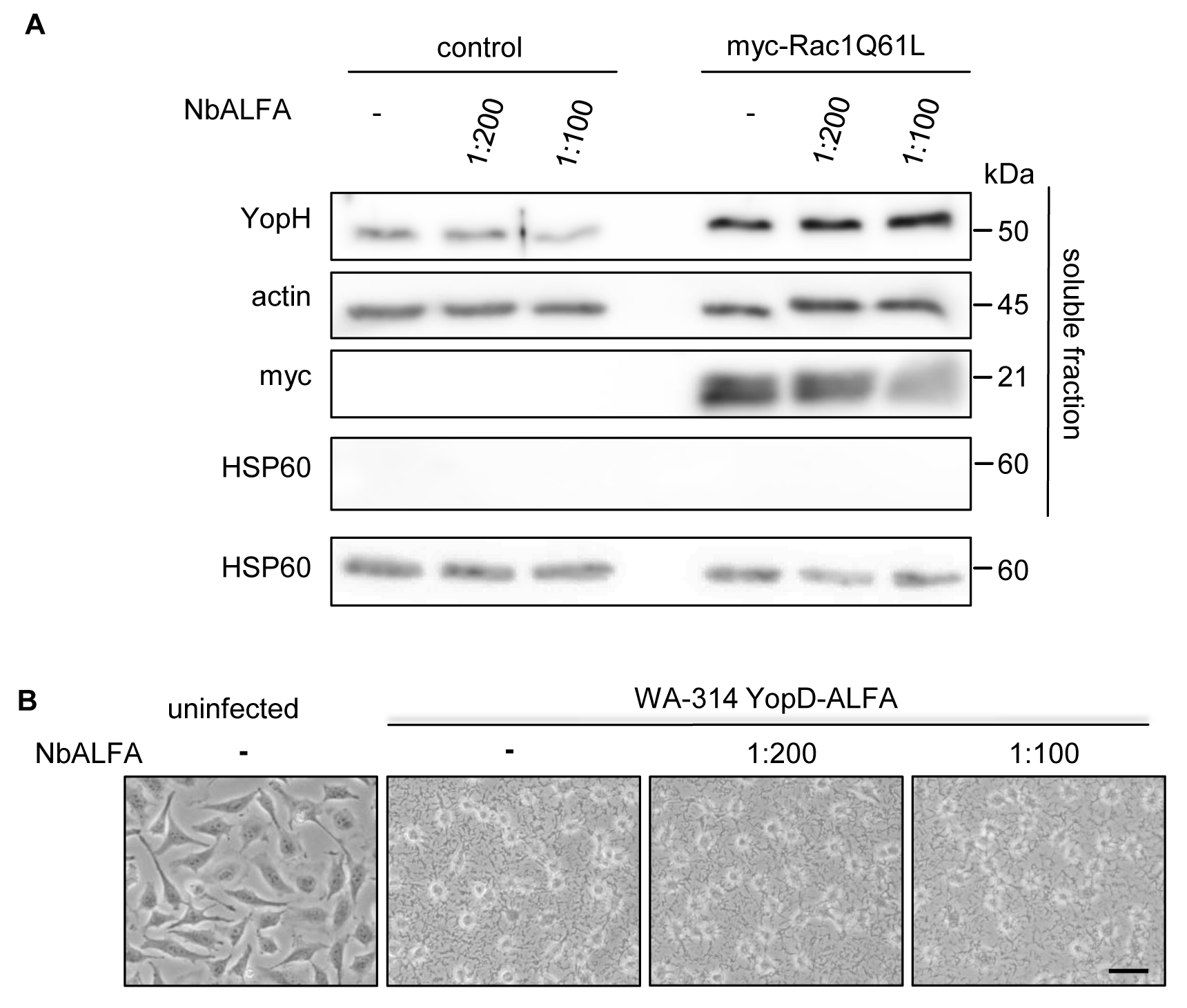

Supplement: S1 Fig — (A) Digitonin assay. Hela cells were infected with WA-314 YopD-ALFA at an MOI of 100. NbALFA-580 was diluted in cell culture medium as indicated. Cells were lyzed with digitonin and resulting supernatants (containing membrane integrated and soluble Yops from host cells) and cell pellets (containing intact bacteria and digitonin insoluble cell components) were analyzed with Western blot for the indicated proteins. YopH serves as marker for effector translocation, myc indicates myc-Rac1QL61 expression, calnexin serves as host cell loading control and HSP60 serves as bacterial loading and lysis control. Data are representative of 2 independent experiments. (B) Cytotoxicity assay. HeLa cells were infected for 1 h with WA-314 YopD-ALFA at an MOI of 100 and imaged by phase contrast microscopy. NbALFA was diluted in cell culture medium as indicated. Depicted are phase contrast images of a representative experiment. Scale bar: 20 μm. (TIF) [file ppat.1010251.s001.tif]

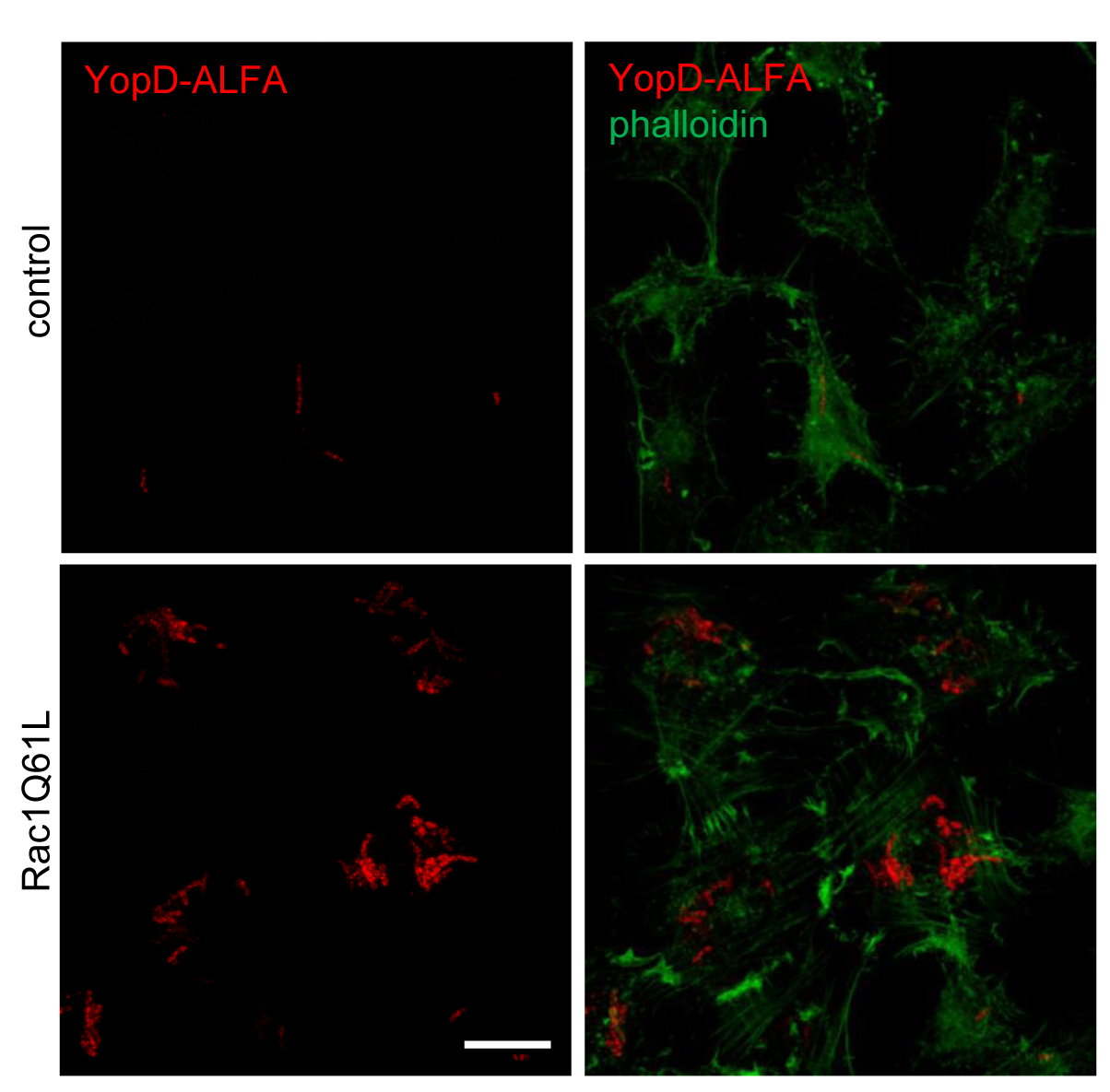

Supplement: S2 Fig — Control or myc-Rac1Q61L transfected Hela cells were infected with WA-314 YopD-ALFA at an MOI of 50 for 50 min, fixed and stained with NbALFA-647 (shown in red) with prior permeabilization of host cell membranes using digitonin. Cells are visualized using phalloidin 488 (shown in green). Scale bar: 20 μm. (TIF) [file ppat.1010251.s002.tif]

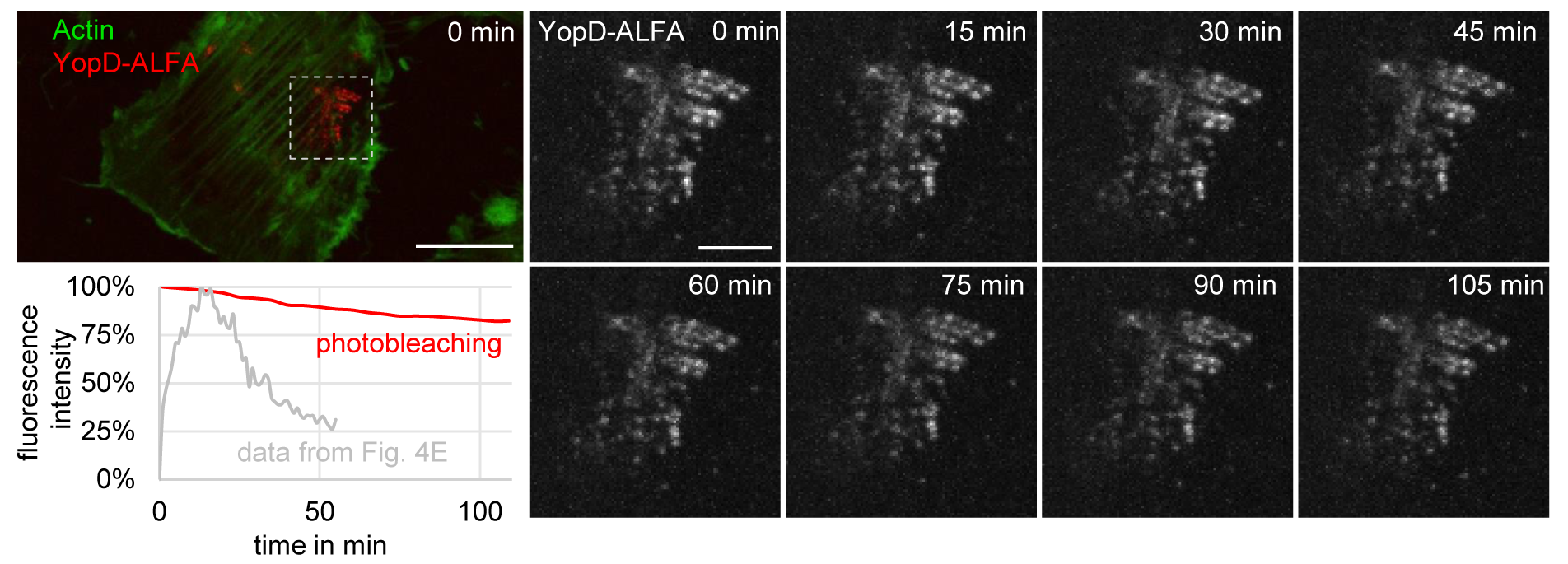

Supplement: S3 Fig — Hela cells were infected at an MOI of 50 for 50 minutes, fixed, permeabilized with digitonin and incubated with NbALFA-580 and phalloidin 488. After addition of cell culture medium, cells were imaged with a spinning disk microscope recording z-stacks every minute for 105 min employing the same imaging conditions as for live cell imaging (e.g. for Fig 4E). The z-stacks for each time point were combined to one image using maximum intensity projection and one image every 15 min is representatively shown. The left panel shows the overview image at 0 min. The boxed region in the overview image shows the area of the video depicted in still frames to the right. Scale bars: 20 μm (overview) and 5 μm (still frames). The relative fluorescence intensity of the NbALFA-580 signals were plotted. For comparison, the live cell imaging data from Fig 4E were included in the graph. (TIF) [file ppat.1010251.s003.tif]

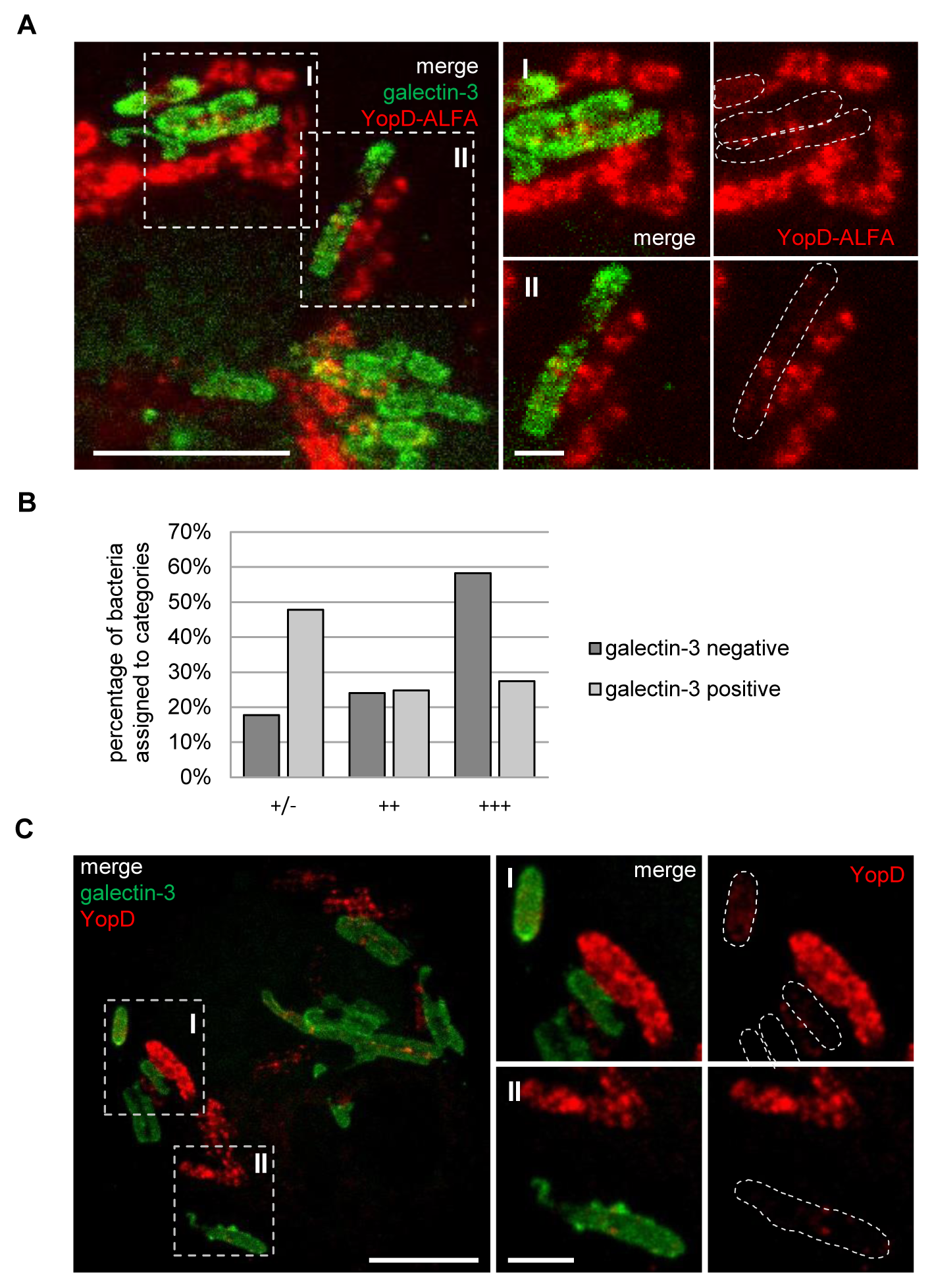

Supplement: S4 Fig — (A) NbALFA staining of translocon associated YopD-ALFA in fixed cells. HeLa cells expressing myc-Rac1Q61L and GFP-galectin-3 were infected with WA-314-YopD-ALFA at an MOI of 50 for 50 min. Cells were fixed, permeabilized with digitonin and stained with NbALFA-647. Scale bars: Overview 10 μm, zoom 2 μm. (B) Semiquantitative categorization of NbALFA signal intensities in galectin-3 positive and negative compartments. Experimental conditions as in A. Bacteria in galectin-3 negative and positive compartments were categorized according to the intensity of YopD-ALFA signals (+/-: no/weak intensity; ++: medium intensity; +++: strong intensity). Total number of bacteria evaluated: n = 400 (C) Immunostaining of translocon associated YopD in fixed cells. Experimental conditions as in A but infection with WA-314 and staining with purified polyclonal anti-YopD antibody. Scale bars: Overview 10 μm, zoom 2 μm. (TIF) [file ppat.1010251.s004.tif]
