# Supplementary material for: Live imaging of Yersinia translocon formation and immune recognition in host cells
Source: PLoS Pathog. 2022 May 23;18(5):e1010251. doi: 10.1371/journal.ppat.1010251 (PMC9173619; doi:10.1371/journal.ppat.1010251)
Supplement: Attachment — Submitted filename: Response to reviewers.pdf [file ppat.1010251.s010.pdf]

*The manuscript implements a novel methodology to study the dynamics of the Yersinia T3SS translocon complex. Reviewers were unanimously enthusiastic about the study. However, all 3 reviewers also raised several concerns regarding technical aspects of the paper that will need to be resolved for a revised MS to be accepted. Especially noteworthy concerns raised by the reviewers center around the questions of whether ALFA tagging/ binding of the nanobody could block translocon function, whether NbALFA loss of signal is definitively reflecting loss of YopD, and why bacteria are found intracellularly in spite of their anti-phagocytic effectors. The study also implicated a role for GBPs and/or galectin-3 in YopD degradation. While these were potentially exciting preliminary findings, the reviewers criticized that the paper didn't provide direct evidence for such a model, e.g. by using cells deficient for GBPs/ Galectin-3. Exploring the biology of YopD 'degradation' further and especially probing for the functional roles of GBPs/ galectins in this process - while certainly desirable - may be beyond the scope of this study and therefore in lieu of more data, the authors could also decide to taper down the biology aspect of their paper, and revise the paper carefully to avoid making unsupported claims.*

***Part I – Summary***

*Please use this section to discuss strengths/weaknesses of study, novelty/significance, general execution and scholarship.*

*Reviewer #1: The manuscript "Live imaging of Yersinia translocon formation and immune recognition in host cells" establishes a new technique using fluorescent nanobodies that recognize an ALFA tag to image the dynamics of translocon complex formation and "degradation" in situ using time lapse fluorescence imaging. The translocon complex is a major constituent of the type 3 secretion system, a molecular device acting as a molecular syringe used by many gram-negative bacterial pathogens. It is crucial for the delivery of bacterial effector proteins into the host cell. Despite its importance the translocon complex cannot easily studied, most likely due to its transient insertion into host cell membranes, and limitations working with its purified components in vitro. Therefore, the approach developed in this manuscript is welcome and has the potential to provide not only important insights into Yersinia cell invasion, however it could also be developed for other bacteria. Overall, the manuscript is written very clearly, and the presented data is well described. Together, it's a great piece that will be very relevant for infection biologists working on secretion machines, such as the T3SS. Nevertheless, there are some overinterpretations in the text (the authors provide often only correlative data, which does not allow causal interpretations)- these issues can easily be amended down-toning some of the biological interpretations. Furthermore, the manuscript could be strengthened by some additional controls that the authors may already have, but not included in the piece, or that could be executed in a reasonable amount of time. I feel that a revised manuscript would become a very strong paper for PLoS Pathogens. Below, I state the points that should be addressed by the authors:*

*Reviewer #2: This manuscript takes a novel and creative approach to studying the mechanisms of Yersinia type III secretion in cells, by tagging a key translocon protein, YopD, with an ALFA tag that can be detected with nano-bodies. using this tag, the authors find that the translocon is*

*formed in a pre-phagosomal compartment, which is not entirely surprising given that Yersinia is an extracellular pathogens and transolcates effectors from the outside of the cell. The authors furthermore use the ALFA-tagged YopD to follow the fate of translocons over time, and find that galectin-3 and GBP-1 are recruited to the phhagosome, followed by loss of the signal, suggesting degradation of the translocon. This is an intriguing approach to studying hte dynamics of translocation with a protein that is essential for the functioning of the translocon, and will likey be applicable to many other such approaches. That being said, I have some questions about the general applicability of these findings to Yersinia biology, given that the function of the Yops is to prevent phagocytosis, and that Yersinia replicates extracellularly.*

*Reviewer #3: In the manuscript "Live imaging of Yersinia translocon formation and immune recognition in host cells," Rudolph et al. use live imaging studies leveraging an ALFA-tag-binding nanobodies to track the timing and localization of Yersinia enterocolitica's YopD as a proxy for translocon formation. They demonstrate that YopD is integrated into translocons just minutes after interaction with host cells. They go on to show that YopD is associated with phosphatidylinositol-4.5-bisphosphate enriched membranes early during infection. At later timepoints they report that YopD signal colocalizes with galectin-3 and guanylate-binding protein-1 (GBP-1), which coincides with a decrease in YopD signal. The authors suggest that this might be due to degradation of the translocon via galectin-3 and/or GBP-1 mediated pathway.*

*The questions tackled by these authors is important: understanding the kinetics and location of translocon formation will provide insights into Yersinia virulence mechanisms. Their live imaging approach is particularly exciting, as it may be adaptable to the study of other bacterial pathogens that encode TTSSs/translocons. Their report that translocon colocalization with Gal-3 and GBP1 coincides with loss of YopD signal, is an intriguing finding. That said, there are several important caveats that should be addressed to fully support the conclusions drawn in this manuscript.*

---

***Part II – Major Issues: Key Experiments Required for Acceptance***

*Please use this section to detail the key new experiments or modifications of existing experiments that should be absolutely required to validate study conclusions.*

*Generally, there should be no more than 3 such required experiments or major modifications for a "Major Revision" recommendation. If more than 3 experiments are necessary to validate the study conclusions, then you are encouraged to recommend "Reject".*

*Reviewer #1: I would suggest experimental data on the two points below:*

*- Figure 1 provides convincing controls to prove that insertion of the ALFA tag does not perturb the translocon activity. However, the rest of the paper focuses on imaging of the translocon insertion, requiring the binding of a nanobody (NbALFA) to the ALFA tag. Therefore, the authors should add some data that the binding of the nanobody does not entirely block translocon*

*function. This could be included in Figure 1.E with an experiment in the presence of the NbALFA. Similar comments could be made for Figure 1.F.*

As suggested by the reviewer, we conducted control experiments to exclude that binding of NbALFA to YopD-ALFA affects translocon function. For this, we infected Hela cells with WA-314 YopD-ALFA in the presence of NbALFA (concentrations same or double as used for live imaging) and assessed translocation of the effector protein YopH by digitonin lysis and Western blotting. This experiment shows that when NbALFA binds to YopD-ALFA during effector translocation, translocation efficiency is unchanged as compared to translocation employing unbound YopD-ALFA. In addition, we show that the cytotoxic effect induced by WA-314 YopD-ALFA is not attenuated in the presence of NbALFA. These data are provided in Fig. S1 A and B and described in the results section, lines 115 - 120.

*- Figure 3 onwards (important to be addressed carefully): The present study relies on observation/quantification of gain and loss of signals through the nanobody binding to the ALFA tag. The authors state that using different intervals of imaging with similar results of their data indicate limited or no photobleaching. This cannot be interpreted as such, as photobleaching relies on different parameters, for example the intensity of the incoming light (instead of the sequence). As this is such an important issue in the manuscript, additional controls should be performed. For example, this could be done by co-straining in an indirect immunofluorescence experiment with NbALFA and an anti YopD antibody at early and again at late infection times after the end of their time-lapse. Failure to detect an antibody signal at later infection stages will provide additional evidence that the NbALFA loss of signal is not due to photobleaching but really to YopD disappearance.*

We thank the reviewer for this important comment. We designed control experiments to evaluate and quantify photobleaching. For this, HeLa cells were infected with WA-314 YopD-ALFA and fixed with paraformaldehyde. Fixed YopD-ALFA was stained with NbALFA-580 and subjected to live imaging employing the same conditions as for NbALFA-580-stained native YopD-ALFA. The fixed YopD-ALFA fluorescence signal decreased by 18 % within 105 min, as compared to a 74 % decrease of the signal in the live imaging experiments during 40 min (Fig. S 3). This indicates that only minor photobleaching occurs during the live imaging time period and a decrease of the fluorescence signal most likely is caused by degradation or dissolution of YopD-ALFA. These results are now included in Fig. S3 and described in the results section, lines 166 - 172.

In addition, we compared and quantified NbALFA- and antibody-mediated fluorescence stainings of YopD-ALFA and YopD, respectively, in fixed cells. The results complement the live imaging data and very clearly show that both, NbALFA- and antibody-mediated fluorescence signals of translocons diminish or disappear at bacteria surrounded by GFP-galectin-3. These results suggests that the translocon protein YopD is degraded or disassembled in association with galectin-3 recruitment but do not formally prove that galectin-3 is functionally involved in YopD disappearance. These results are now included in Fig. S4 and described in the results section, lines 224 – 232.

*Reviewer #2: 1. Injection of Yops E and H blocks phagocytosis. If the translocon is functioning appropriately, how is Yersinia entering the cell? I recognize that some portion of Yersinia are indeed inside the cell, but these would presumably be bacteria whose T3SS was not functioning as well. It is important to know how the conclusions here impact the extracellular bacteria that are presumably the pool of replicating ones.*

It has been shown in previous work that the antiphagocytic effect of pathogenic yersiniae is not an all or nothing effect but depends on infection time, uptake mechanism and host cell type. Upon start of a host cell infection, a large fraction of the bacteria that contact the cell, whether lacking or expressing the T3SS, are taken up. This process is either mediated by bacterial surface proteins like Invasin or YadA (e.g., in epithelial cells) or surface opsonization by antibodies or complement (e.g., in phagocytes) (Visser et al. PMID: 7790071; Black et al. PMID: 10931345; Grosdent et al. PMID: 12117925; Zhang et al. PMID: 18591234). When the cell infection progresses, an increasing number of bacteria expressing a T3SS resists phagocytosis and stays extracellularly. In addition, both virulent and avirulent yersiniae have been reported to be taken up by an Invasin-mediated process in a so called prevacuolar compartment (Bahnan et al. PMID: 26275291, Sarantis et al. PMID: 22341461) and only in this compartment the translocon can be formed (Nauth et al. PMID: 30586431).

*2. The authors conclude from the loss of YopD fluorescence signal that it is being degraded. Is it fair to conclude that? Could recruitment of galectin and GBPs to the phagosome and bacterial membrane interfere with the ability of the nanobody to bind to YopD? Is there a way to measure the levels of YopD by western blotting of digitonin-treated cells which should provide access to the extracellular YopD pool?*

This is an important comment and similar to the major issue raised by reviewer #1. Detection of YopD by Western blotting in digitonin-soluble fractions of infected cells was demonstrated by us previously (see Nauth et al. PMID: 30586431). However, this method does not allow to draw conclusions about the fate of YopD in individual translocons. We therefore compared and quantified NbALFA- and antibody-mediated fluorescence stainings of YopD-ALFA and YopD, respectively, in fixed cells. The results complement the live imaging data and very clearly show that both, NbALFA- and antibody mediated fluorescence signals of translocons diminish or disappear at bacteria surrounded by GFP-galectin-3. These results suggests that the translocon protein YopD is degraded or disassembled in association with galectin-3 recruitment but do not formally prove that galectin-3 is functionally involved in YopD disappearance. These results are now included in Fig. S4 and described in the results section.

*3. Can the authors perform these studies in the absence of Gbps (GbgpChr3-/- cells for example?) a prediction of their model is that the translocon should not be degraded in these cells.*

This is, of course, a very interesting question, but we feel that the required experiments are beyond the scope of this study. Accordingly, we have toned down the statements about the functional role of GBP1 in translocon degradation as suggested by the reviewer and editor.

*4. Given that YopD itself is translocated, how do the authors know that the YopD they are imaging is part of a functional translocon, rather than YopD that is being translocated into the cytosol of the target cell? This seems important to address to be able to support the conclusion being reached. If the translocon is degraded in a GBP-dependent manner, presumably in the absence of GBPs, that degradation would not occur, and there should be more translocated effectors, which could be measured by beta-lactamase assay.*

This comment is important and similar to the issue raised by reviewer #3 (see also answer to reviewer #3).

In our previous study we showed that the YopD signals investigated in the present study colocalize with signals of YopB and the needle tip protein LcrV by STED microscopy and are in the expected distance of around 100 nm to signals of the basal body marker GFP-YscD in individual injectisomes (Nauth et al. PMID: 30586431). In parallel, we showed that the YopD signals are embedded in a PIP2 enriched membrane compartment by STED microscopy. Thus, the imaging data are fully compatible with translocons localized at the tip of the injectisome and embedded in the host membrane.

In addition, it has previously been shown by Feeley et al. (PMID: 28193861), using *Y. pseudotuberculosis* mutants, that the translocon is required for recruitment of Galectin-3. In the current manuscript, we confirm this finding (Fig. 4B) and show that galectin-3 is recruited only to bacteria that previously displayed a YopD-ALFA signal (Fig. 4C). We therefore think that the specific recruitment of Galectin-3 to YopD-ALFA positive bacteria is another strong indication that the observed YopD-ALFA signals represent membrane-inserted translocons at the tip of injectisome needles.

As mentioned above we decided not to include experiments involving GBP knockout cell lines.

*Reviewer #3: Overall the manuscript is written well, however there is important details that are lacking throughout the manuscript that make the experimental design/data/figures hard to understand. For example: there was limited information about the strain background. Was the tagged YopD complementing a deltaYopD mutant or was there an endogenous copy? I also had to dig for the MOIs, conditions for the secretion assay, beta-lactamase reporter, etc. More detail in the results section is absolutely required to enhance the manuscript.*

We are sorry for not having provided these informations in the current manuscript. We now included more details of the experimental procedures in the results section. E.g. we clarified that WA314-YopD-ALFA mutant was generated by CRISPR-Cas assisted recombineering leading to modification of the endogeneous copy of YopD to preserve physiological expression levels (lines 88 – 92). The beta-lactamase-, cytotoxicity- and secretion assays are now explained in more detail in the results section (lines 97 - 99; 105 – 110; 110 – 113). The MOIs are indicated throughout the figure legends, which we would like to keep for better readability of the main text.

*The major advance of this manuscript is the ability to potentially visualize the formation, localization and overall dynamics of the translocon. Since this is a new technique that the entire manuscript hinges on, there needs to be additional evidence to indicate that the authors are indeed visualizing the translocon. One way this could be addressed is via biochemical isolation of phagosomal membranes, followed by western blot analysis probing for YopD at key time points. This type of experiment would support their conclusion that they are indeed visualizing YopD/the translocon (enrichment of the protein in the membrane fraction should coincide with visualization of ALFA-tag-binding nanobodies in their time-lapse movies). In addition, further modulating of the system would help support the conclusion that they are the visualizing YopD/the translocon. For example, the authors state that cells expressing myc-Rac1Q61L strongly increases translocon expression. Does expression of myc-Rac1Q61L (compared to controls) increase YopD signal in the live imaging system?*

These are important comments and similar to the issue raised by reviewer #2.

We agree with the reviewers that it is important to be sure that the assay does indeed visualize translocon-associated YopD-ALFA. However, we believe that the current manuscript and our previous study provide several lines of evidence that this is the case:

1. In our previous study we showed that the YopD signals investigated in the present study colocalize with signals of YopB and the needle tip protein LcrV by STED microscopy and are in the expected distance of around 100 nm to signals of the basal body marker GFP-YscD in individual injectisomes (Nauth et al. PMID: 30586431). In parallel, we showed that the YopD signals are embedded in a PIP2 enriched membrane compartment by STED microscopy. Thus, the imaging data are fully compatible with translocons localized at the tip of the injectisome and embedded in the host membrane.

2. It has previously been shown by Feeley et al. (PMID: 28193861), using *Y. pseudotuberculosis* mutants, that the translocon is required for recruitment of Galectin-3. In the current manuscript, we confirm this finding (Fig. 4B) and show that galectin-3 is recruited only to bacteria that previously displayed a YopD-ALFA signal (Fig. 4C). We therefore think that the specific recruitment of Galectin-3 to YopD-ALFA positive bacteria is another strong indication that the observed YopD-ALFA signals represent membrane-inserted translocons at the tip of injectisome needles.

3. We have shown before in the same experimental setup that activation of Rac1 activity increases both, the number of translocon positive yersiniae and overall effector Yop translocation rate (Wolters et al. PMID: 23803609, Nauth et al. PMID: 30586431). This correlation suggests that the higher level of translocated effectors is generated by a higher number of actively translocating bacteria. To clarify the role of constitutively active Rac1L61, we now included imaging data for control and myc-Rac1L61 expressing HeLa cells infected with WA-314 YopD-ALFA. The overview image clearly shows the increased number of translocon forming bacteria in the Rac1L61 expressing cells. We describe this experiment in the results section (lines 154 – 156) and Fig. S2.

*The authors find YopD signal colocalizes with galectin-3 and guanylate-binding (GBP-1), which coincides with a decrease in YopD signal. The authors suggest that this might be due to degradation of the translocon via galectin-3 and/or GBP-1 mediated pathway. This is potentially very interesting and the dependency is testable. It would greatly enhance their findings if the authors can conclude that YopD half-life is impacted by galectin-3 and GBP-1. Using gal-3 and GBP-1 knockdowns or knockouts and visualizing the spaciotemporal localization of YopD via live cell imaging/western blot would help draw such conclusions. In addition, modulating the system (e.g. inhibiting vacuole acidification or macrophage activation) and measuring how YopD signal changes over time would give additional support. This set of experiments will help give deeper insights into the relationship of Gal-3 and GBP-1 to YopD. Without this data this is simply an observation that the Yersinia translocon colocalizes with markers of membrane damage.*

We thank the reviewer for this important suggestion. However, as stated above, we feel that analysing the functional role of galectin-3 and GBP-1, e.g., using knockout cells, is beyond the scope of this study. Accordingly, we have toned down the statements about the functional role of GBP1 in translocon degradation as suggested by the reviewer and editor.

---

***Part III – Minor Issues: Editorial and Data Presentation Modifications***

*Please use this section for editorial suggestions as well as relatively minor modifications of existing data that would enhance clarity.*

*Reviewer #1: -Figure 2.B – The STED data is very good and relates very well to previous work of the authors using translocon specific antibodies. I am wondering how to interpret the dot in the middle of the bacterium, as it appears that this translocon is not fully in focus. Therefore, the authors should include a 3D STED dataset or they should use a dataset where the translocons out of focus do not complicate the interpretation of the data.*

We thank the reviewer for this suggestion. We included a 3D STED dataset (in Fig. 2B) to improve the visualization of internal and translocon-associated YopD-ALFA.

*-Figure 3. The authors state that T3SS translocon pore is inserted after PIP2 enrichment of the vacuolar membrane. I am wondering whether this recruitment does already require some T3SS effector injection. As PIP2 is normally strongly enriched at the plasma membrane (it is well depicted on Figure 3C), and then it gets depleted during phagosome formation, one wonders when does the T3SS really start to deliver effectors… the authors should discuss possible limitations of the fluorescence signals and also relate this to previous work from the Grinstein lab.*

We agree with the reviewer that this aspect requires some more explanation. As shown by Sarantis et al. (PMID: 22341461) also *Yersinia* devoid of a T3SS gets taken up into the PIP2-enriched prevacuole compartment, indicating that no effectors are required for this process.

The membrane forming this compartment is derived from the plasma membrane and therefore a priori enriched with PIP2. We found translocons, which are absolutely required for translocation, exclusively in bacteria either residing in the prevacuolar compartment or after they passed this compartment. We therefore conclude that translocation most likely only starts after uptake of the bacteria into the prephagosome/prevacuole. It can be speculated whether effector translocation then mediates an arrest of the further uptake process and establishes a stable niche for survival of the bacteria in the prevacuole. In any case, we also regularly observed translocon positive bacteria later in the uptake process, i.e., in closed phagosomes devoid of PIP2.

We have now toned down all statements suggesting that changes in PIP2 concentration or phaphatidylinositol reorganisations preceed translocon formation.

*Figure 4G: The GBP signal does not label all phagosomes/vacuoles that contain bacteria- this is interesting and the authors may want to discuss this issue. Movie relating to 4G and figure: at later time points, there are unspecific signals of the nanobody channel- what are these signals? They are not translocons that are carried away to some other compartments (for example for degradation?)? Please discuss this issue.*

As also commented by the reviewer, in the live imaging experiments we repeatedly observed that not all galectin-3-positive compartments also recruited GBP-1 during the recorded time intervals. We do not know the reason for this presently. However, we cannot exclude the possibility that recruitment might still occur at even later time points.

We currently do not know what the observed aggregates of NbALFA, which are seemingly unspecifically attached to the cell or free in the medium, represent. However, we never observed translocon associated signals being carried away from the bacteria.

*The authors use the myc-Rac1Q61L cell line which provides a boost in translocon insertion and the observed infection dynamics. It has been used by the authors before, and it would be good to explain this again in this work, so that the reader can follow why it was used.*

This issue was also raised by reviewer #2. We now included a comparison of WA-314 YopD-ALFA translocon formation in control and Rac1L61 expressing HeLa cells. The overview image clearly shows the increased number of translocon forming bacteria in the Rac1L61 expressing condition. We describe the reasoning for expressing myc-Rac1Q61L in the infected cells on basis of this experiment in the results section and Fig. S2.

*Important general comment: The paper tends to over-interpret some observations without providing additional experiments needed to prove such statements. Of course, it would be possible to address this with extra experiments, however, as the work appears as a proof-of-concept method work, one could down-tone the writing, and suggest experiments to really proof "causal" relationships in the future. A number of examples for this are*

**Lines 166-169: It is not clear whether PIP2 insertion is required for translocon insertion from the shown experiments. The data is a simple correlation. Here, experiments with inhibitors or kinases and phosphatases would be required to really mark the point. Nevertheless, this could simply be discussed.*

We changed the respective section to avoid over-interpretation. We have now omitted all statements claiming that changes in PIP2 concentration or phaphatidylinositol reorganisations are required for translocon formation.

*Similar issues *Lines 192-193: here again, two fluorescence signals are correlated, but a causal link is beyond the acquired data. This can easily be discussed. *Lines 207-209: the shielding of GBP to avoid antibody binding is speculative and would require more controls- again, this could be suggested and elaborated in the discussion.*

In our opinion, these statements are already formulated very cautiously. However, we have added a paragraph in the discussion emphasizing that further experiments, e.g. with knockout cell lines, are needed to establish a causal relationship between galectin-3/GBP-1 recruitment and translocon degradation.

*Reviewer #2: (No Response)*

*Reviewer #3: (No Response)*
